# PTPN22 Is a Critical Regulator of Fcγ Receptor–Mediated Neutrophil Activation

**DOI:** 10.4049/jimmunol.1600604

**Published:** 2016-11-02

**Authors:** Sonja Vermeren, Katherine Miles, Julia Y. Chu, Donald Salter, Rose Zamoyska, Mohini Gray

**Affiliations:** * Medical Research Council/University of Edinburgh Centre for Inflammation Research, Queen’s Medical Research Institute, Edinburgh EH16 4TJ, United Kingdom;; † Institute for Genetics and Molecular Medicine, Edinburgh EH4 2XU, United Kingdom; and; ‡ Institute of Immunology and Infection Research, Ashworth Laboratories, University of Edinburgh, Edinburgh EH9 3FL, United Kingdom

## Abstract

Neutrophils act as a first line of defense against bacterial and fungal infections, but they are also important effectors of acute and chronic inflammation. Genome-wide association studies have established that the gene encoding the protein tyrosine phosphatase nonreceptor 22 (PTPN22) makes an important contribution to susceptibility to autoimmune disease, notably rheumatoid arthritis. Although PTPN22 is most highly expressed in neutrophils, its function in these cells remains poorly characterized. We show in this article that neutrophil effector functions, including adhesion, production of reactive oxygen species, and degranulation induced by immobilized immune complexes, were reduced in *Ptpn22*^−/−^ neutrophils. Tyrosine phosphorylation of Lyn and Syk was altered in *Ptpn22*^−/−^ neutrophils. On stimulation with immobilized immune complexes, *Ptpn22*^−/−^ neutrophils manifested reduced activation of key signaling intermediates. *Ptpn22^−/−^* mice were protected from immune complex–mediated arthritis, induced by the transfer of arthritogenic serum. In contrast, in vivo neutrophil recruitment following thioglycollate-induced peritonitis and in vitro chemotaxis were not affected by lack of PTPN22. Our data suggest an important role for PTPN22-dependent dephosphorylation events, which are required to enable full FcγR-induced activation, pointing to an important role for this molecule in neutrophil function.

## Introduction

Neutrophils are the most abundant peripheral blood leukocytes in humans. As part of the innate immune system, they provide an immediate response to infection or injury. Neutrophils are rapidly activated by a variety of stimuli, including bacterial peptides, complement, and immune complexes (ICs). Autoimmune diseases, including rheumatoid arthritis (RA), are associated with the generation of ICs that accumulate in synovial fluid or are deposited on articular cartilage surfaces. They engage and activate neutrophils via FcγRs (1, 2). Severe inflammation follows neutrophil degranulation, releasing a plethora of degradative enzymes and other inflammatory mediators (3). The ensuing release of reactive oxygen species (ROS) and proteases degrades articular cartilage, whereas secreted chemokines attract further immune cells into the joint, driving chronic inflammation (4). Thus neutrophilic inflammation forms a crucial part of the inflammatory response, which needs to be resolved in a timely manner to minimize host damage.

Protein tyrosine phosphatase nonreceptor 22 (PTPN22) is a leukocyte-restricted phosphatase associated with an increased risk in a range of autoimmune diseases, notably RA. The single missense nucleotide polymorphism (SNP) C1858T encoding an R620W substitution is the single most important non-MHC gene contributor to RA susceptibility, and the second most important for juvenile idiopathic arthritis according to candidate gene and genome-wide association studies (5, 6). Although expression of PTPN22 is highest in the neutrophil (7), its function in these myeloid cells remains largely unknown. In T cells, PTPN22 has been shown to suppress TCR signaling, for instance, by dephosphorylating key tyrosine residues within the activation loops of the Src family kinases (SFKs) Lck and Fyn and the TCR adapter Zap-70. At least in T cells, PTPN22 cooperates with the C-terminal Src kinase; their physical interaction is critical to their synergistic regulatory function. On a protein level, the disease-associated R620W variant (R619W in the mouse) affects one of four proline-rich regions in the C terminus of PTPN22. This disrupts PTPN22 binding to C-terminal Src kinase (8, 9).

The K/B×N serum transfer arthritis model of arthritis is induced by administration of arthritogenic serum from arthritic KRN × NOD donors. This bypasses the need for an adaptive immune system–driven break in self-tolerance. It results in a transient, but rapidly evolving, inflammatory arthritis that reproduces many of the hallmarks of RA (10, 11). In combination with a range of experimental approaches, including genetic lineage depletion and reconstitutions, this disease model has helped to elucidate the important contribution of innate immune cells, notably neutrophils, to the effector phase of RA (12–14).

In this article, we present an analysis of PTPN22 function in the neutrophil, concentrating on FcγR signaling owing to its prominent role in autoimmune diseases. By performing functional assays with isolated neutrophils from PTPN22-deficient mice and by analyzing inflammation in K/B×N serum transfer arthritis, we demonstrate that PTPN22 regulates FcγR neutrophilic inflammation.

## Materials and Methods

Unless otherwise stated, materials were obtained from Sigma.

### Abs

Abs directed against phosphotyrosine (PY1000), phospho–spleen tyrosine kinase (Syk) (Y525/526), phospho-pan Src (Y527 and Y416), phospho-Akt (S473), phospho-p38 (T180, Y182), and phospho-Erk (T202, Y204) were from Cell Signaling Technology. Anti-Syk (clone 5F5) and anti-Lyn (clone LYN-01) were from BioLegend. Anti-Ly6G (clone RC6-8C5) was obtained from R&D Systems. Anti-BSA and anti-lactoferrin Abs were from Sigma and an Ab against β-COP was a gift from Nick Ktistakis (The Babraham Institute, Cambridge, U.K.). HRP-conjugated secondary Abs were from Santa Cruz Biotechnology and Bio-Rad. Fluorescently conjugated Abs for flow cytometry were obtained from eBioscience (F4/80, GR1), BioLegend (CD11b, CD11a, CD16/32, Ly6G, CD62L, CD19), and BD (Ly6C).

### *PTPN22* mouse model

Generation of the *PTPN22* mouse has been previously described (15). Experimental mice were housed in individually ventilated cages in a specific and opportunistic pathogen–free small animal barrier unit at the University of Edinburgh. All animal work was approved by United Kingdom Home Office Project license PPL60/4567.

### Analysis of peripheral blood

Peripheral blood was sampled from the superficial temporal vein into sodium citrate. Leukocytes were stained using fluorescently conjugated Abs, mixed with flow-check fluorospheres (Beckman Coulter) to determine cell numbers, and RBCs were lysed (BD FacsLyse). Samples were analyzed by flow cytometry using an LSR Fortessa (BD).

### K/B×N serum transfer arthritis model

The K/B×N serum transfer model was induced using pooled arthritogenic serum as previously described (10, 11, 16). Mice were scored for 20 d. Each limb was scored as follows: 0, normal; 1, erythema or swelling in a single digit; 2, erythema and swelling in two or more joints; 3, swelling of the entire paw, including the hock joint. The sum of the score for each limb (giving a maximum possible score of 12) was taken as a measure of the extent of arthritis at that time point. Formalin-fixed samples were prepared for histological examination by decalcification in EDTA. The 3-μm sections were stained with H&E. Immunostaining for Ly6G was performed according to standard procedures without Ag retrieval, using a secondary HRP-coupled Ab followed by a diaminobenzidine reaction. Analysis of histological specimens occurred in a blinded fashion by a histopathologist, using an Olympus BX51 microscope. Images were acquired with a Micropublisher 3.3RTV camera and Q Imaging software using ×2, ×20, and ×40 objectives.

### Thioglycollate-induced sterile peritonitis

Sterile peritonitis was induced by injection of matured 4% Brewer’s thioglycollate (BD) at a concentration of 20 ml/kg. Mice were sacrificed 3 h after induction and peritoneal cells harvested following a peritoneal flush. Cells were stained with fluorescently conjugated Abs and mixed with flow-check fluorospheres (Beckman Coulter) to determine cell numbers, before being analyzed by FACS using the LSR Fortessa (BD).

### Neutrophil purification

Neutrophils were isolated from bone marrows of 12- to –14-wk-old sex- and age-matched mice using discontinuous Percoll gradients (GE Healthcare Amersham, Uppsala, Sweden) as previously described (17, 18), using endotoxin-free reagents throughout.

### Immobilized ICs and immobilization of adhesive proteins

For immobilized ICs, dishes were coated overnight at 4°C with endotoxin and fatty acid–free BSA in PBS (100 μg/ml), blocked with 1% fat-free milk in PBS, and incubated with rabbit anti-BSA (1:2000). Control surfaces were treated identically, but not incubated with Ab. Some assays were carried out with insoluble ICs, which had been generated as described (19). Human fibrinogen (150 μg/ml), polyRGD (20 μg/ml), or, as a control, heat-inactivated FCS was adsorbed onto tissue culture–grade plastics overnight at 4°C and for 3 h at room temperature, respectively. All surfaces were extensively washed with PBS prior to performing any assays.

### ROS production assays

ROS production was measured by chemiluminescence in a Synergy H1 plate reader (BioTek) using a luminol-based assay in luminescence grade 96-well plates (Nunc; Thermo Scientific) essentially as previously described (20). Measurements were started immediately following cell stimulation, and light emission was recorded. Data output was relative light units per second.

### Chemotaxis assays

For chemotaxis assays, neutrophils were resuspended in HBSS supplemented with 15 mM HEPES pH 7.4 and 0.05% fatty acid and endotoxin-free BSA. Dunn chambers were assembled as described (21), and chemotaxis assays were carried out with MIP2 (R&D Systems) as chemoattractant. Cells were monitored by time-lapse imaging for 30 min using an inverted RMDIB microscope (Leica) equipped with temperature-controlled chamber, automated stage (Prior), Orca camera (Hamamatsu), and Micromanager image acquisition software (Fiji). Paths of all individual neutrophils were tracked using the manual tracking plug-in into ImageJ. Tracks were subsequently analyzed using the Chemotaxis Tool (Ibidi) plug-in into Image J.

### Transendothelial migration assays

Transendothelial migration (TEM) assays were carried out as previously described (22). Briefly, 6.5-μm Transwell inserts with 3-μm polycarbonate membranes (Corning Costar U.K.) were coated overnight with 2 μg/ml fibronectin and seeded with 5 × 10^4^ bEnd5 cells grown in DMEM supplemented with 10% heat-inactivated FBS (both Life Technologies) as described. Confluent bEnd5 cells were stimulated for 16 h with 5 nM TNF-α, and 5 × 10^5^ neutrophils were added into washed inserts that had been placed into wells of 24-well plates in the presence of 0, 1, or 3 nM MIP2 and allowed to migrate toward the chemoattractant. Transmigrated neutrophils were labeled for GR1, and 8 randomly chosen fields of view per 24 wells were photographed for counting (×20 magnification using an Evos cell imaging system [AMG]).

### Adhesion assays

Neutrophils were allowed to adhere to immobilized IC or blocked surfaces at 37°C in 96-well plates. Dishes were then subjected to rapid orbital shaking for 30 s before contents were flicked sharply out of wells prior to cell fixation. Following extensive washing, four random images were taken from each well for analysis of cell spreading (×20 magnification; Evos system).

### Degranulation assays

Gelatinase granule release after plating neutrophils onto an immobilized IC-coated surface, or following stimulation with fMLF and cytochalasin B, was detected by in-gel zymography as previously described (20). Lactoferrin release was assayed by making use of an Ab to human lactoferrin that had previously been shown to cross-react with mouse protein essentially as described (23, 24).

### Analysis of protein phosphorylation

Neutrophils were plated onto immobilized IC-coated or control-treated dishes for stimulation. For analysis of phosphorylation of protein kinase B (PKB), Erk, and p38 MAPK, this was done as previously described (20, 25). For analysis of Lyn and Syk, nonadherent cells and scraped, adherent cells were combined for lysis in ice-cold 100 mM NaCl, 30 mM HEPES pH 7.4, 20 mM NaF, 1 mM EGTA, 1% Triton X-100, 1 mM benzamidine, 10 μg/ml aprotinin, 1 mM PMSF, 1 mM Na_3_VO_4_, as well as mammalian protease inhibitor mixture and phosphatase inhibitor mixture 2. After pelleting detergent-insoluble material, proteins of interest were immunoprecipitated using protein G agarose and Abs as indicated. Carefully washed beads were boiled in sample buffer, and immunoprecipitated proteins and lysate controls were separated by SDS-PAGE. Proteins were transferred to Immobilon membrane (Millipore) for Western blotting, using Abs as indicated.

### Cytokine-release assays

Cytokine-release assays were carried out using insoluble HSA–anti-HSA ICs as stimulation. Insoluble ICs were prepared by titrating the point of equivalence between Ag and Ab and monitoring the point of equivalence by measuring the absorbance at 450 nM as previously described (19). Neutrophils were stimulated as indicated and cultured in round-bottom 96-well plates (Corning) in Dulbecco’s PBS supplemented with Ca^2+^ and Mg^2+^, 1 g/l glucose, and 4 mM sodium bicarbonate in a humidified atmosphere at 37°C and 5% CO_2_. After 6 h, supernatants were harvested for cytokine analysis by ELISA (R&D research) according to the manufacturer’s instructions.

### Statistical analysis

For kinetic experiments, the area under the graph was used for analysis. Where data met the assumptions for parametric tests, two-tailed Student *t* tests were applied. Otherwise, the nonparametric Mann–Whitney test was used. A *p* value < 0.05 was considered statistically significant.

## Results

**FIGURE 1.**
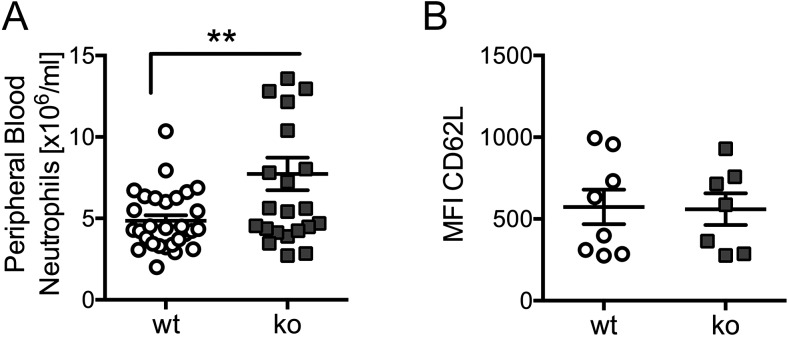
*Ptpn22*^−/−^ mice have mild neutrophilia. (**A**) Blood samples from 17 *Ptpn22^−/−^* and 22 matched control mice were labeled for CD11b and Ly6G and analyzed by flow cytometry. Data (means ± SEM) were pooled from four separate experiments. (**B**) Cell surface L-selectin was analyzed in unstimulated neutrophils from blood samples of eight WT and 7 *Ptpn22^−/−^* mice. Neutrophils were gated for analysis of CD62L staining; mean fluorescence intensity (MFI) values are plotted. **p < 0.01. ko, knockout.

*Ptpn22^−/−^* mice have already been described. In line with previous reports, we found them to be fertile and without overt deleterious or autoimmune phenotype (15, 26). *Ptpn22*^−/−^ mice were characterized by mild peripheral blood neutrophilia (Fig. 1A), complementing previous observations of increased B and T cell numbers in *Ptpn22*^−/−^ mice (26). To test whether this mild neutrophilia was a result of inadequate activation of *Ptpn22*^−/−^ neutrophils, we analyzed cell surface L-selectin/CD62L on freshly drawn peripheral blood neutrophils from control and *Ptpn22*^−/−^ mice. The findings were similar, suggesting that the neutrophil activation status was not affected by the absence of PTPN22 (Fig. 1B).

### PTPN22 regulates neutrophil adhesion to immobilized ICs

**FIGURE 2.**
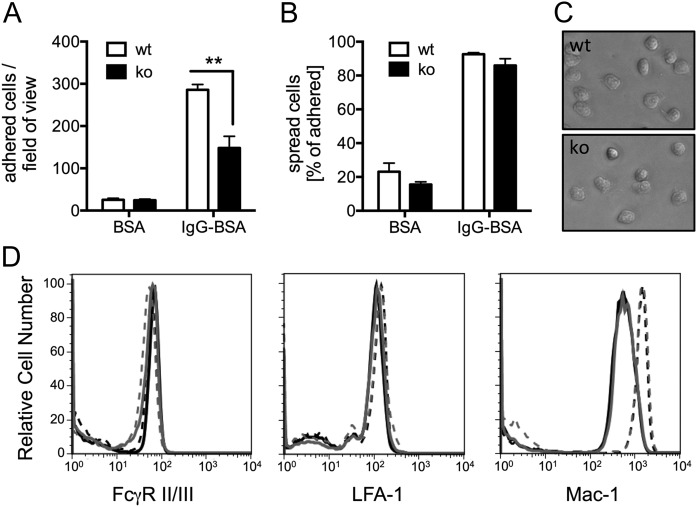
*Ptpn22*^−/−^ mice have a defect in adhering to immobilized ICs. Neutrophils isolated from bone marrows of *Ptpn22^−/−^* [knockout (ko)] and matched WT control mice were allowed to adhere for 20 min to wells that had been coated with immobilized ICs (IgG-BSA) or BSA as control, followed by washing and fixing. Adherent and spread cells were counted in four randomly photographed fields of view of each well. (**A**) Total number of all adhered cells. (**B**) Percentage of spread cells out of total. (A and B) Pooled data (means ± SEM) from three separate experiments are presented. (**C**) Representative examples of *Ptpn22^−/−^* and matched control neutrophils were allowed to adhere to immobilized ICs for 20 min. (**D**) Surface FcγRII/III, LFA-1, and Mac-1 of purified bone marrow–derived neutrophils from control and *Ptpn22*^−/−^ mice were analyzed by flow cytometry. Neutrophils were or were not stimulated with fMLF at 37°C before being labeled with FITC-conjugated anti-GR1 and PE-conjugated anti-FcγRII/III, anti–Mac-1, or anti–LFA-1. GR1-positive cells were gated, and PE staining was measured. Results were analyzed using FlowJo software. Black lines, WT neutrophils; gray lines, *Ptpn22*^−/−^ neutrophils; full lines, unstimulated cells; broken lines, stimulated cells. Experiments were performed with cells from four animals per genotype, and representative examples are shown. See Supplemental Fig. 1 for a graphical representation of all experiments. **p < 0.01.

Given the documented role of PTPN22, neutrophils, and ICs in autoimmune-driven inflammatory arthritis, we analyzed IC-induced effector functions with purified, bone marrow–derived neutrophils. When plating the cells onto surfaces that had been coated with immobilized ICs, fewer *Ptpn22^−/−^* than wild-type (WT) control neutrophils were found to adhere firmly (Fig. 2A). In contrast, both *Ptpn22^−/−^* and WT control neutrophils were equally able to spread, once attached to the immobilized ICs (Fig. 2B, 2C). Similar results were obtained with neutrophils that had been allowed to bind to immobilized ICs for a shorter time (10 min; not shown). Neutrophils of both genotypes displayed very similar levels of surface FcγRII/III (Fig. 2D). Because FcγRs and β2 integrins are known to cross-talk extensively (27, 28), we also characterized cell surface integrins. We observed no difference in cell surface expression of the common leukocyte β2 integrins LFA-1/CD11a and noted a similar increase in cell surface Mac-1/CD11b following stimulation of *Ptpn22^−/−^* and WT control neutrophils with fMLF (Fig. 2D; Supplemental Fig. 1). These data suggest that reduced adhesion of *Ptpn22*^−/−^ neutrophils was not due to reduced cell surface FcγR or integrin receptor density.

### PTPN22 regulates adhesion- and FcγR-dependent ROS formation

**FIGURE 3.**
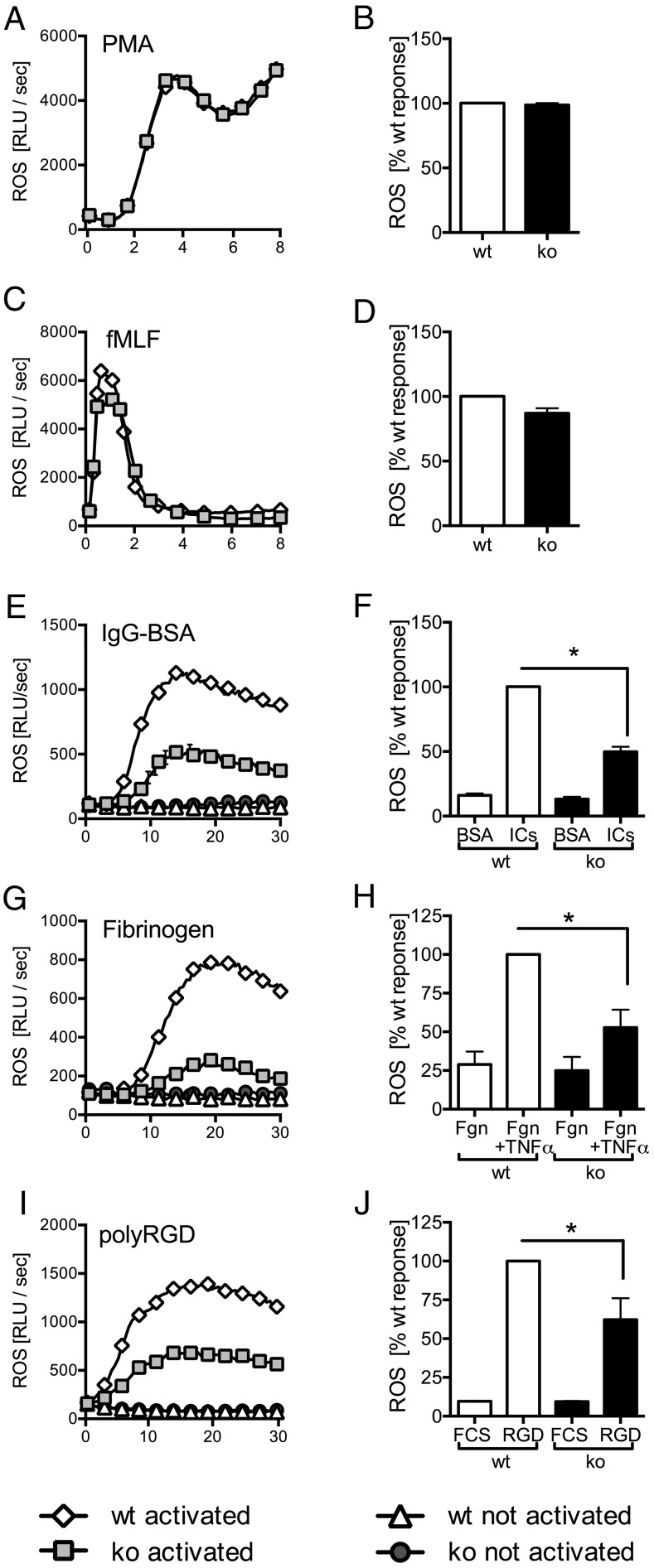
*Ptpn22*^−/−^ neutrophils are characterized by impaired immune receptor–induced ROS production. Neutrophils isolated from bone marrows of *Ptpn22^−/−^* [knockout (ko)] and matched WT control mice were used to characterize ROS production following stimulation with PMA (**A** and **B**), with fMLF (**C** and **D**), or following plating of cells into wells coated with immobilized IC (IgG-BSA) or, as a control, BSA (**E** and **F**), into fibrinogen-coated wells in the presence (stimulated) or absence (control) of TNF-α (**G** and **H**), or into wells coated with the synthetic integrin ligand poly-Arg-Gly-Asp (polyRGD) or, as a control, heat-inactivated FCS (**I** and **J**). Data shown (means ± range) are representative individual experiments (A, C, E, G, and I). Total light emissions (means ± SEM) expressed as percentage of the response obtained with stimulated control neutrophils pooled from a minimum of three separate experiments (B, D, F, H, and J). *p < 0.05. RLU, relative light unit.

FcγR engagement initiates specific neutrophil effector functions, including ROS production and degranulation. To test whether *Ptpn22^−/−^* neutrophils were able to generate ROS, we stimulated cells with the phorbol ester PMA (Fig. 3A, 3B) or with a formylated peptide (fMLF) (Fig. 3C, 3D). We noted no differences between genotypes. In contrast, ROS formation by *Ptpn22^−/−^* neutrophils that had been plated onto immobilized ICs was significantly lower than that seen with WT control neutrophils (Fig. 3E, 3F). In line with well-established cross-talk between FcγRs and integrins (27, 28), a similarly reduced generation of ROS was also observed when neutrophils were plated on the β2 integrin ligand fibrinogen in the presence of TNF-α (Fig. 3G, 3H) or onto the synthetic pan integrin ligand poly-Arg-Gly-Asp (Fig. 3I, 3J). In conclusion, *Ptpn22^−/−^* neutrophils were characterized by reduced ROS production following FcγR and integrin-dependent stimulations.

### PTPN22 regulates FcγR-dependent neutrophil degranulation

**FIGURE 4.**
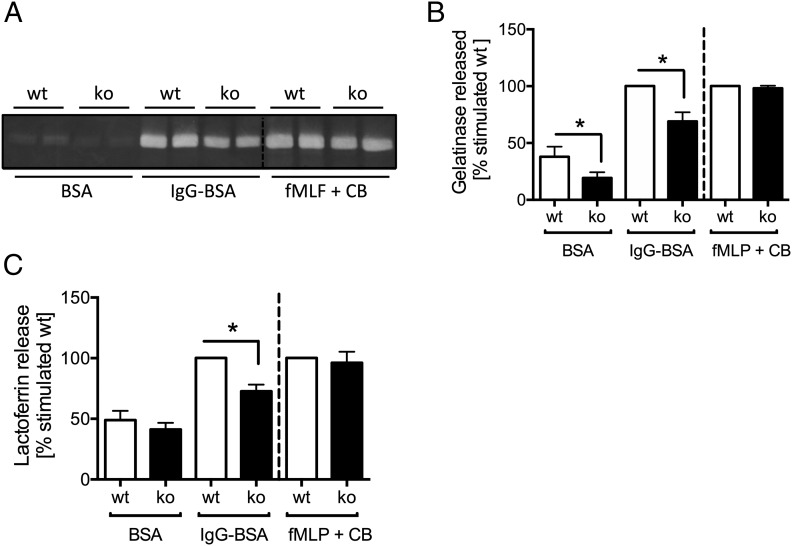
*Ptpn22*^−/−^ neutrophils are characterized by reduced IC-induced degranulation. (**A** and **B**) Gelatinase release by control (wt) and *Ptpn22^−/−^* [knockout (ko)] neutrophils plated into wells coated with immobilized ICs (IgG-BSA) or, as a control, BSA, or that had been stimulated by fMLF in the presence of cytochalasin B (fMLF + CB) was assessed by in-gel zymography. A representative experiment (A) and pooled data (means ± SEM) from five separately performed experiments (B) are presented. (**C**) Lactoferrin release by control and *Ptpn22^−/−^* neutrophils plated into wells coated with immobilized ICs (IgG-BSA) or, as a control, BSA, or that had been stimulated by fMLF in the presence of cytochalasin B (fMLF + CB) was assayed by ELISA. Pooled data from three separate experiments are presented. Data presented are normalized to activated WT. *p < 0.05.

We next tested the ability of *Ptpn22*^−/−^ neutrophils to degranulate by measuring the release of lactoferrin and gelatinase, components of neutrophil secondary and tertiary granules, respectively. As observed with ROS production, *Ptpn22^−/−^* neutrophils degranulated less efficiently than controls when plated onto immobilized ICs, but not when they were stimulated with fMLF in the presence of cytochalasin B (Fig. 4). These data argue that PTPN22 is required for optimal neutrophil effector functions involved in generating an inflammatory environment following stimulation of FcγR (and integrins), and likely functioning in intracellular signaling events downstream of receptor engagement.

To address whether PTPN22 may also be involved in cytokine production, we analyzed release of TNF-α, MIP2 (a murine equivalent of IL-8), and IL-1β by neutrophils that had been stimulated with ICs. The extent of cytokine release triggered by IC stimulation of neutrophils detected in these assays was very low, and any reductions observed with *Ptpn22^−/−^* neutrophils did not reach significance (Supplemental Fig. 2).

### PTPN22 functions to activate signaling processes downstream of FcγRs

**FIGURE 5.**
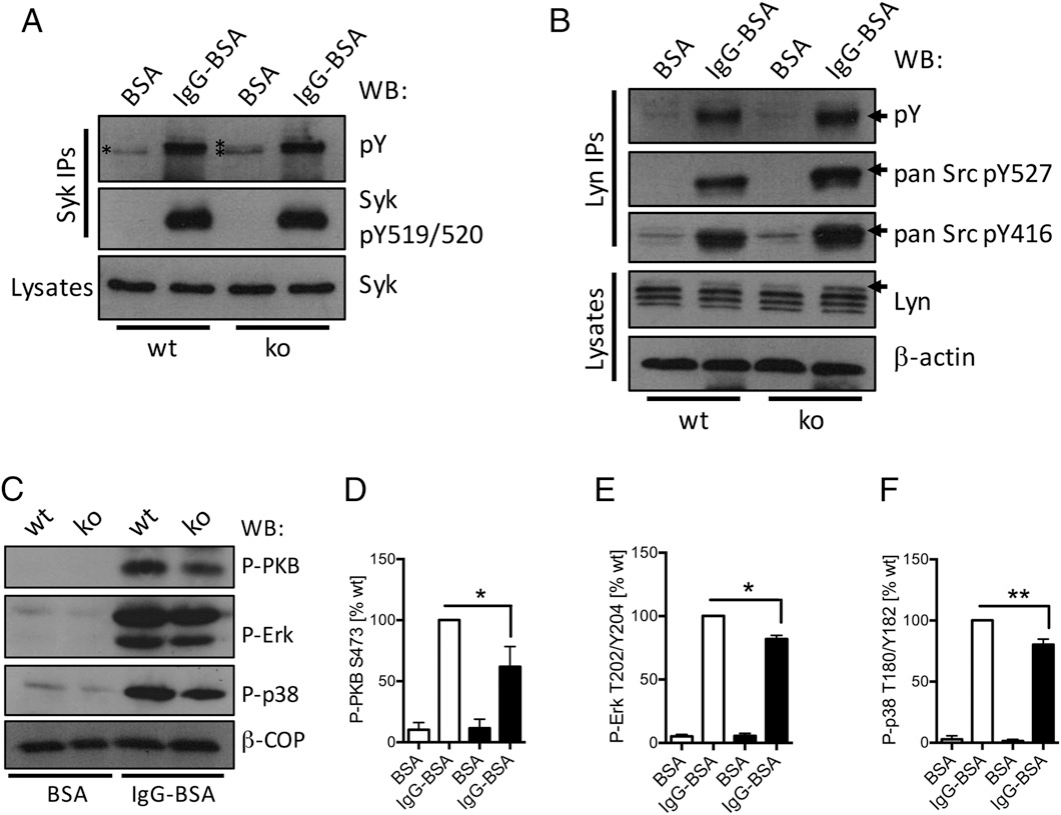
Neutrophil PTPN22 regulates Lyn and Syk tyrosine phosphorylation, which affects the activity status of many signaling cascades. Neutrophils isolated from bone marrows of *Ptpn22^−/−^* [knockout (ko)] and matched WT control mice were allowed to adhere to immobilized ICs (IgG-BSA) or, as a control, BSA for 12 min. (**A** and **B**) Lysates were prepared as detailed in *Materials and Methods* for Western blotting, using the indicated Abs; in addition, Syk (A) or Lyn (B) was immunoprecipitated for analysis of phosphotyrosine as well as probing with Abs detecting specific tyrosine phosphorylations in Syk/SFK proteins as indicated. Representative samples from at least three separately performed experiments are shown. (**C**–**F**) Lysates were prepared and subjected to immunoblotting with Abs specific for phospho-PKB, phospho-Erk, and phospho-p38 MAPK (C–F). Blots were quantitated using Image J software. (C) Representative blots. (D–F) Densitometry from five separate experiments (means ± SEM) was normalized to activated controls. *p < 0.05, **p < 0.01.

PTPN22 is a protein tyrosine phosphatase. Signaling downstream of FcγRs (and integrins) involves significant tyrosine phosphorylation. Receptor-proximal key signaling proteins regulated include the major neutrophil SFKs Lyn, Hck, and Fgr, as well as Syk. We analyzed tyrosine phosphorylation of Syk immunoprecipitated from control and *Ptpn22^−/−^* lysates from neutrophils that had been plated onto immobilized ICs (IgG-BSA) or onto BSA (Fig. 5A). Mock-stimulated *Ptpn22^−/−^* neutrophils were characterized by increased Syk tyrosine phosphorylation (221.4 ± 16.8% compared with control) as well as a second, slightly slower migrating band (indicated by * in Fig. 5A) in comparison with WT controls. No differences were apparent between Syk tyrosine phosphorylation of activated WT and *Ptpn22^−/−^* neutrophils. Probing with an Ab that specifically recognizes the activating phosphotyrosine residues Y519/Y520 in mouse Syk revealed no differences between genotypes, with no signal in basal control or *Ptpn22^−/−^* neutrophils. This finding suggested that Syk tyrosine residue(s) other than Y519/Y520 are dephosphorylated either directly or indirectly by PTPN22 in the neutrophil.

Lyn has been shown to act as an inhibitory SFK that phosphorylates Syk in B cells (29, 30). We therefore analyzed Lyn in control and *Ptpn22^−/−^* neutrophils. As a result of differential splicing, Lyn is known to run as a doublet (31); however, at least with the Ab we used, Lyn migrated as three distinct bands in basal and stimulated control cells as well as in basal *Ptpn22^−/−^* cells. In lysates from stimulated *Ptpn22^−/−^* neutrophils, we noticed a fourth Lyn band (Fig. 5B, arrow). Given the short time-frame of the stimulation used, this was likely caused by a posttranslational modification of Lyn. We therefore immunoprecipitated Lyn for analysis with phosphorylation-specific Abs. This analysis revealed increased total tyrosine phosphorylation of *Ptpn22^−/−^* neutrophils (150.1 ± 6.2% compared with control) and slight increases for both the activating Y527 residue (124.5 ± 6.1%) and the inhibitory Y416 residue (118 ± 2.7%). In addition, phosphorylated Lyn from *Ptpn22*^−/−^ neutrophils migrated slower than Lyn from controls. This was particularly apparent for the phospho-Y527 (Fig. 5B, arrows). Taken together, our observations suggested that PTPN22 regulates Lyn phosphorylation in the neutrophil.

To further address the implications of PTPN22-mediated dephosphorylation events, we also analyzed the activity status of some key signaling intermediates that are activated downstream of SFK/Syk signaling following activation of FcγRs. We probed for phospho-PKB (also known as Akt), a read-out for the activity status of phosphoinositide 3-kinase, phospho-Erk, and phospho-p38 MAPK. All of these signaling intermediates were found to be mildly hypophosphorylated in *Ptpn22^−/−^* neutrophils (Fig. 5C–F), indicating that many signaling events downstream of FcγR engagement are influenced by PTPN22 in the neutrophil. Finally, phosphotyrosine blots of lysates from control and *Ptpn22^−/−^* neutrophils that had been plated onto BSA or immobilized ICs showed reduced tyrosine phosphorylation in basal and activated *Ptpn22*^−/−^ lysates (Supplemental Fig. 3). Together, these in vitro data suggested that PTPN22 phosphatase performs activating dephosphorylation events in the neutrophil.

### *Ptpn22^−/−^* mice are protected from K/B×N serum transfer arthritis

**FIGURE 6.**
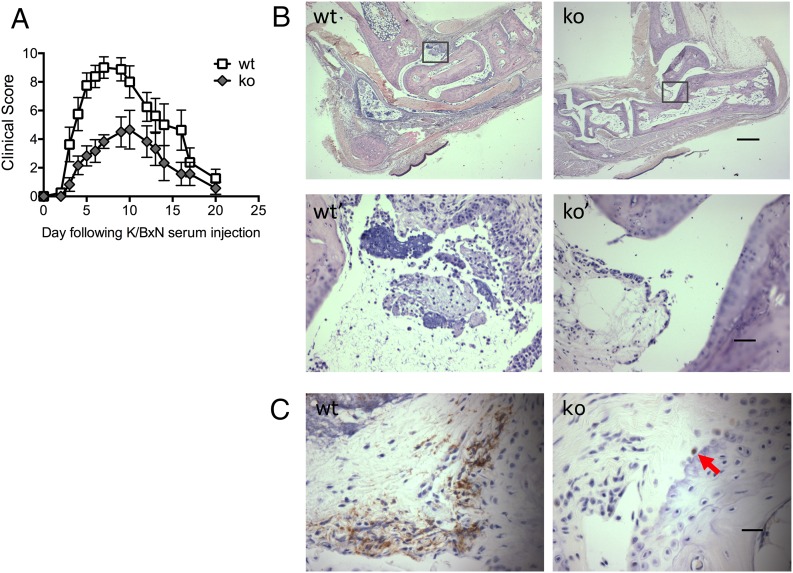
Ptpn22^−/−^ mice are protected from K/B×N arthritis. K/B×N serum transfer arthritis was induced in *Ptpn22^−/−^* [knockout (ko)] and matched control (wt) mice. (**A**) Clinical scores (means ± SEM) of the experimental groups were plotted over 20 d. Four experiments were carried out with five to eight animals in each experimental group; a representative experiment is shown; experimental groups behaved significantly differently; *p* = 0.02. (**B**) Wax sections of decalcified joints were H&E stained to visualize joint erosion and leukocyte infiltration on day 5 after serum transfer. Representative examples from sections obtained with right rear joints of arthritogenic serum–injected control (wt) and *Ptpn22*^−/−^ (ko) mice are shown (scale bar, 500 μm); panels wt′ and ko′ are higher power images of the boxed areas in panels wt and ko (scale bar, 50 μm). (**C**) Wax sections of decalcified joints were stained with an Ab specific for Ly6G and counterstained with hematoxylin to visualize neutrophil infiltration at day 2 following administration of arthritogenic serum. An arrow in *Ptpn22^−/−^* points out a rare neutrophil (scale bar, 25 μm).

Given the association of the human PTPN22 SNP, C1858T, with RA, we tested whether the in vitro observations were relevant in vivo. K/B×N serum was administered to control and *Ptpn22*^−/−^ mice to induce serum transfer arthritis. *Ptpn22*^−/−^ mice were significantly protected from inflammatory joint disease compared with age- and sex-matched WT controls. This protection was independent of the sex of the recipient animals, and the trend was observed with various doses of arthritogenic serum (Fig. 6, Table I). *Ptpn22*^−/−^ mice were characterized by slower progression of the disease and also reduced overall severity (Fig. 6A). The clinical observations in the *Ptpn22*^−/−^ mice were confirmed in histological sections taken near the height of inflammation (on day 5), which showed a reduced inflammatory cell infiltrate and few joint erosions when compared with WT controls (Fig. 6B). Very few neutrophils were apparent in ankle joint sections at this time point. Therefore, neutrophil infiltration was further evaluated at an earlier time point (day 2), when neutrophil influx was expected to be maximal. Neutrophils in joint sections were visualized by their distinct morphology and by Ly6G staining (Fig. 6C, red arrows). In contrast to controls and in keeping with the milder clinical disease, very few neutrophils were apparent in ankle joints of *Ptpn22^−/−^* mice.

**Table I.** *Ptpn22^−/−^* mice are protected from K/B×N arthritis

Recipient	*n*	Treatment	Mean Time to Peak (d)	Mean Maximal Score (± SD)	Time at Peak Intensity (d)	Median Clinical Score
WT female	9	75 μl on day 0	6.2	8.6 (± 1.9)	2	3.8
ko female	9	75 μl on day 0	6.8	5.0 (± 1.9)	3.4	2.3
WT female	4	100 μl on day 0, 100 μl on day 2	5.8	10.25 (± 1.6)	4	8.25
ko female	3	100 μl on day 0, 100 μl on day 2	7	6.0 (± 1.7)	3.7	3.0
WT male	6	100 μl on day 0	4.2	9.8 (± 1.6)	2.3	4.0
ko male	6	100 μl on day 0	6.7	6.5 (± 2.9)	3.3	3.3

Irrespective of the gender of the recipient and of disease induction with a single injection or repeat injections with arthritogenic serum, *Ptpn22^−/−^* mice were characterized by slower disease progression and reduced peak amplitude.

ko, knockout.

### PTPN22 does not regulate neutrophil migration or recruitment

**FIGURE 7.**
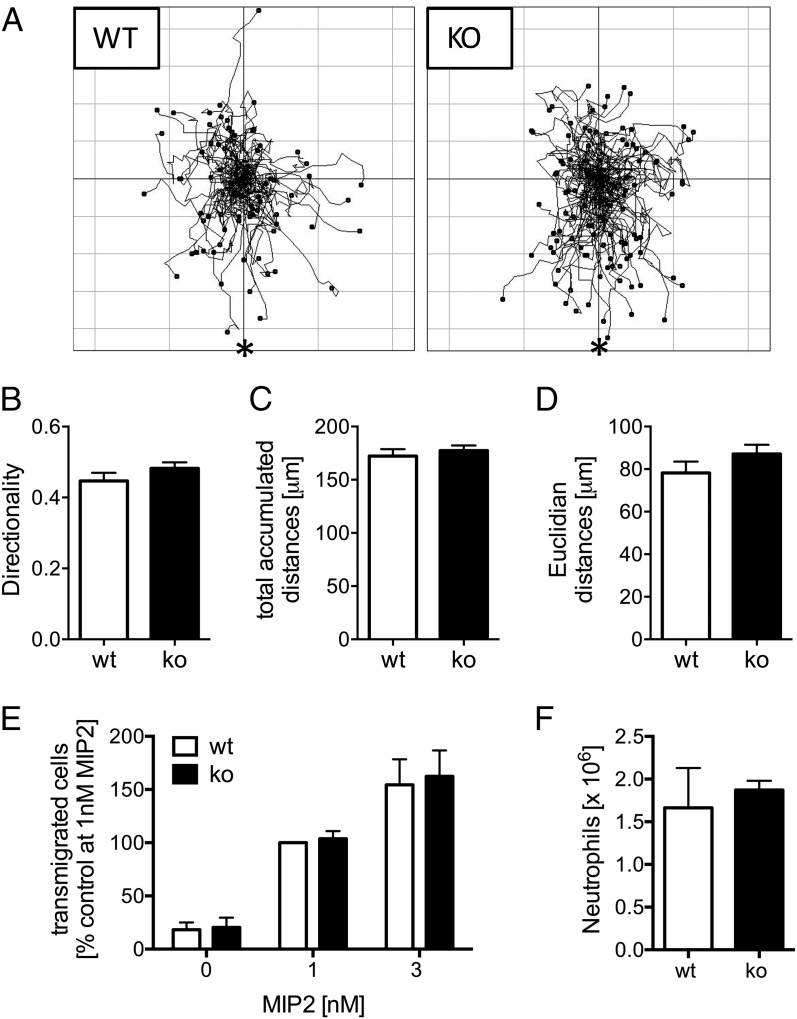
Neutrophil chemotaxis and recruitment are not affected in Ptpn22^−/−^ mice. (**A**–**D**) Neutrophils isolated from bone marrows of *Ptpn22^−/−^* [knockout (ko)] and matched WT control mice were allowed to chemotax toward 10 nM MIP2 in Dunn chambers. Cell migration was monitored by time-lapse imaging, and individual cells were tracked using a cell tracking plug-in into Image J. (A) Tracks obtained in experiments carried out with three separate preparations of bone marrow–derived neutrophils analyzed were pooled for these spider plots. The source of chemoattractant is indicated (*). (B–D) The coordinates for the tracks shown in (A) were analyzed using statistics features of the Ibidi chemotaxis plug-in into Image J, as detailed in *Materials and Methods*. Parameters presented (means ± SEM) are directionality (B), total accumulated (C), and Euclidian distances (D); differences were not statistically significant. (**E**) *Ptpn22^−/−^* (ko) and control (wt) neutrophils were allowed to migrate toward indicated concentrations of MIP2 in Transwells supporting a monolayer of TNF-α–stimulated mouse endothelial cells. Data (means ± SEM) expressed as percentage of control cells migrating toward 1 nM MIP2 from two pooled experiments carried out with separately prepared neutrophils are presented. (**F**) Peritonitis was induced in *Ptpn22*^−/−^ (ko) and WT control mice by injection of thioglycollate-containing broth. Peritoneal flushes were analyzed to enumerate numbers (means ± SEM) of peritoneal neutrophils. A representative experiment performed with six controls and five *Ptpn22^−^*^/^*^−^* mice is shown. Differences were not statistically significant.

Neutrophils have been shown to organize their own recruitment to the inflamed joint in K/B×N serum transfer arthritis. This recruitment is regulated by a series of chemokines and cytokines that are released by neutrophils and other synovial cells and that mediate recruitment of consecutive waves of neutrophils to the inflamed joint (32, 33). Because neutrophil migration into the inflamed joint is an absolute requirement for the generation of inflammation in this model (12–14), we analyzed chemotaxis of *Ptpn22^−/−^* and control neutrophils. We observed no differences in terms of Euclidian or total accumulated distances traveled or indeed in the chemotactic directionality (the ratio between the Euclidian and the total accumulated distance) in Dunn chamber chemotaxis toward MIP2, indicating that *Ptpn22^−/−^* neutrophils undergo normal chemotaxis (Fig. 7A–D). We further addressed whether the absence of PTPN22 might affect TEM, a key step required to enable neutrophils to leave the bloodstream. TEM was assessed using an in vitro Transwell chemotaxis assay, in which neutrophils migrated through a Transwell membrane supporting a monolayer of TNF-α–stimulated murine endothelial cells. *Ptpn22^−/−^*-deficient neutrophils migrated through these activated endothelial cells as efficiently as WT controls (Fig. 7E), suggesting that PTPN22 does not regulate neutrophil TEM either. Finally, to test more definitively leukocyte recruitment in vivo, we used the thioglycollate-induced sterile peritonitis model. Leukocyte recruitment was analyzed at an early time point after induction of peritonitis, when neutrophil recruitment peaks ahead of the recruitment of other leukocytes (34). Again, and in line with the in vitro observations above, there was no detectable defect in neutrophil recruitment to the inflamed peritoneum of *Ptpn22^−/−^*-deficient mice (Fig. 7F). These observations demonstrate that PTPN22 does not regulate neutrophil chemotaxis, TEM, or recruitment per se. Rather, PTPN22 influences the activation of neutrophils in response to ICs.

## Discussion

Our data show a significant protection of *Ptpn22*^−/−^ mice from IC-driven K/B×N serum transfer arthritis. This model tests the effector phase of arthritis, which depends on neutrophil activation for its clinical expression. Protection was observed with both males and females under a number of experimental conditions (Fig. 6, Table I). In contrast to our results, Wang et al. (35) and Maine et al. (36) noted no protection from joint protection using the K/B×N model in *Ptpn22*^−/−^ mice. We suspect that these discrepancies may be the result of differences in the experimental conditions. Researchers in both studies induced arthritis by injecting larger amounts of arthritogenic serum than we did, inducing a severe and prolonged arthritis. In contrast, we aimed for submaximal transient arthritis, which would enable the detection of subtle differences between experimental cohorts. It is conceivable that the degree of neutrophil stimulation induced in these studies overrode the protection afforded by the lack of PTPN22. In addition, it is possible that differences in housing conditions would lead to variations in the microbiome of the mice used for the different studies. The gastrointestinal microbiota has recently been recognized to regulate immunity and inflammatory responses to in vivo challenges (37). Interestingly, K/B×N arthritis depends heavily on the microbiome, with the T cell–independent effector phase of K/B×N serum transfer arthritis being regulated by gut microbiota (38, 39).

In vitro, with *Ptpn22^−/−^* neutrophils, we observed normal chemotaxis and TEM toward MIP2. In vivo, normal levels of neutrophil recruitment in the thioglycollate-induced model of peritonitis were also apparent in *Ptpn22*^−/−^ mice. This finding argues against a role for PTPN22 in neutrophil migration or recruitment (Fig. 7). In contrast, few neutrophils were observed in the ankle joints of *Ptpn22*^−/−^ mice following the induction of K/B×N arthritis (Fig. 6). One explanation for this difference is that alternative mechanisms may be used by neutrophils migrating to different sites. Neutrophils have been shown to promote their own recruitment to inflamed joints in the K/B×N serum transfer arthritis model, by generating chemokines and inciting other cells to do the same (32, 33). Therefore, an alternative explanation may be that *Ptpn22^−/−^* neutrophils were less efficient at promoting a suitable inflammatory environment to drive forward further waves of neutrophil recruitment, as seen with the K/B×N serum transfer arthritis. In vitro, neutrophil activation by immobilized ICs was defective, whereas stimulation induced by fMLF or PMA was not (Figs. 3, 4). This observation indicates that PTPN22 is likely to be preferentially involved in inflammation, induced following integrin- and/or FcγR-dependent neutrophil activation.

Our observations are reminiscent of recent reports with Syk and SFK triple knockout (ko) neutrophils that provided complete protection from K/B×N serum transfer arthritis (40, 41). In these reports, Syk, along with Lyn, Hck, and Fgr, was shown to be dispensable for neutrophil recruitment. Yet they played a crucial role in FcγR-/integrin-mediated neutrophil effector functions, including the generation of chemokines that are required to generate inflammation (40, 41). We analyzed IC-induced chemokine generation by *Ptpn22*^−/−^ neutrophils (Supplemental Fig. 2). Overall, cytokine secretion in these experiments was low, and any differences between genotypes did not reach significance. It is likely such differences will be more pronounced with inflammatory neutrophils, such as those within the synovial fluid of the arthritic joint, than with unprimed, bone marrow–derived neutrophils. It will be interesting to address these issues in more depth in the future.

Of note, our data suggest that tyrosine phosphorylation of both Lyn and Syk is affected in *Ptpn22^−/−^* neutrophils (Fig. 5). We do not yet know the identity of the affected phosphorylation sites, nor whether these were direct events. It will be interesting to identify the exact nature of neutrophil PTPN22 substrates in the future. Given that the protection from K/B×N serum transfer arthritis afforded by *Ptpn22^−/−^* mice was larger than that of any individual SFK (Hck, Fgr, or Lyn) (41), it is interesting to speculate that PTPN22 may contribute to regulating several SFKs, as well as other potential substrates. Such a function would be in keeping with our current understanding of PTPN22 function in T cells, in which PTPN22 has been shown to dephosphorylate tyrosine residues within the SFKs Lck and Fyn; ZAP-70, a Syk family protein; and immune receptor tyrosine-based activation motifs in TCR-associated proteins (26, 42, 43). Although these substrates are not expressed in neutrophils, some of their family members are.

The findings presented in this article contrast sharply with observations by others who analyzed the role of PTPN22 in lymphocytes. In T cells, PTPN22 functions as a negative regulator (44). For instance, TCR signaling and cell adhesion, mediated by Rap-dependent integrin inside-out signaling, were increased in *Ptpn22^−/−^* mice (15). The activating role of PTPN22 downstream of FcγRs might be specific to the neutrophil, suggesting that PTPN22 expression might individually modulate various leukocytes that contribute to inflammation.

A missense SNP in PTPN22 (PTPN22-C1858T) predisposes carriers to a number of autoimmune diseases, most notably RA, systemic lupus erythematosus, and type 1 diabetes (43, 45, 46). However, it protects against Behçet’s disease and bowel inflammation secondary to Crohn’s disease. Both of these diseases are characterized by significant neutrophil-mediated chronic inflammation (47, 48). Our understanding of PTPN22 function and biochemistry, in particular in the neutrophil, lags behind studies of its function in T cells. Indeed, the effects of the R620W mutation are still being debated, with suggestions varying between the mutation being activating, inactivating, and a hypomorph (45). Moreover, observations in the mouse do not always tally with those in humans, with studies in human cells split between those suggesting the R620W variant is a gain of function (43, 49) and others suggesting that it is a loss of function (50). In contrast, most studies conducted in the mouse are more suggestive of a loss of function mutation (51, 52). Furthermore, genetic background, as well as the presence of other, potentially as yet unidentified susceptibility alleles, may play a role. R619W *Ptpn22* knockins largely replicated the phenotype of *Ptpn22*^−/−^ lymphocytes, suggestive of a loss of function mutation. One study suggested R619W PTPN22 is prone to degradation (52). However, in a separately derived knockin, protein stability was not impaired and mice kept on a mixed 129 × C57BL/6 background, which is more prone to autoimmunity than pure C57BL/6, developed mild, spontaneous autoimmune disease (51).

In ascribing to PTPN22 an activating function, our work is suggestive, at least in the neutrophil, of a gain of function with R620W PTPN22. In the context of the results described in this article, it is hard to explain how a loss of function mutation would trigger enhanced inflammation in immune receptor–dependent contexts, such as the K/B×N model for arthritis. It will be very interesting to test this hypothesis in the future by analyzing neutrophils derived from an R619W *Ptpn22* point mutation knockin mouse. As noted above, such mice have already been generated, but their analysis to date has been restricted to cell types other than the neutrophil (51, 52). One recent study analyzed effector functions in human neutrophils derived from healthy donors and from RA patients who were or were not homozygous carriers of the SNP (53). Although that study did not test any IC-dependent events, the authors reported that neutrophils from homozygous carriers generate increased ROS, release more calcium in response to other stimulations, and undergo faster TEM than those from matched control donors, in general agreement with the notion that R620W PTPN22 acts as a gain of function mutation in neutrophils.

## Supplementary Material

Data Supplement
