# Supplementary material for: PTPN22 Is a Critical Regulator of Fcγ Receptor–Mediated Neutrophil Activation
Source: J Immunol. 2016 Nov 2;197(12):4771–9. doi: 10.4049/jimmunol.1600604 (PMC5136470; doi:10.4049/jimmunol.1600604)
Supplement: Data Supplement [file JI_1600604.zip › JI_1600604_Supplemental_Figures_1.pdf]

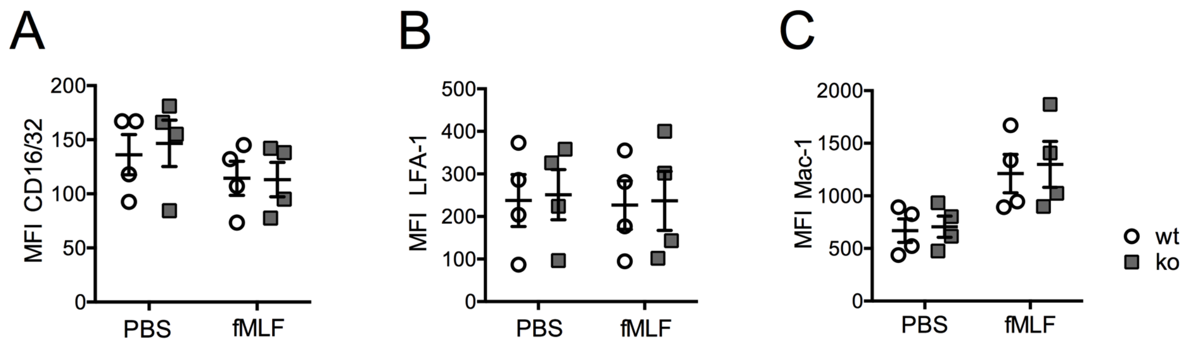

Supplemental figure 1. Cell surface FcγR and integrins are not affected in PTPN22-deficient neutrophils. Surface FcγRII/III, LFA-1 and Mac-1 of purified bone marrow derived neutrophils from control and *Ptpn22*$^{-/-}$ mice were analyzed by flow cytometry. Neutrophils were or were not stimulated with fMLF at 37°C before being labelled with FITC-conjugated anti-GR1 and PE-conjugated anti-FcγRII/III, anti-Mac1 or anti-LFA1. GR1 positive cells were gated and PE staining was measured. Results were analysed using FlowJo software. MFIs are plotted.

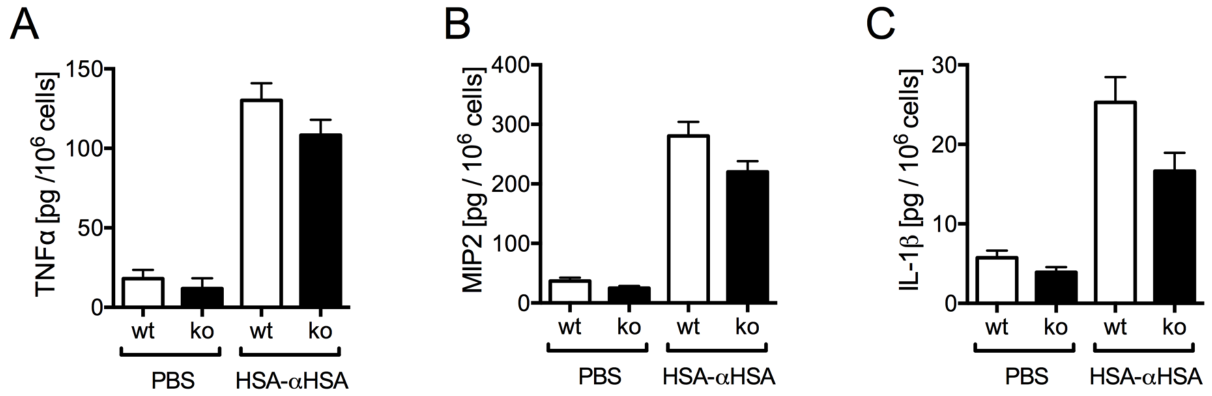

Supplemental figure 2. Cytokine production by IC-stimulated neutrophils. Bone marrow derived control and *Ptpn22*$^{-/-}$ neutrophils were stimulated with 20μg/ml insoluble ICs (HSA-αHSA) and incubated for 6 hours before culture supernatants were harvested for analysis by ELISA for released TNFα (D), MIP2 (E) and IL-1β (F). Data shown were pooled from 4-5 separately performed experiments; differences between genotypes did not reach statistical significance.

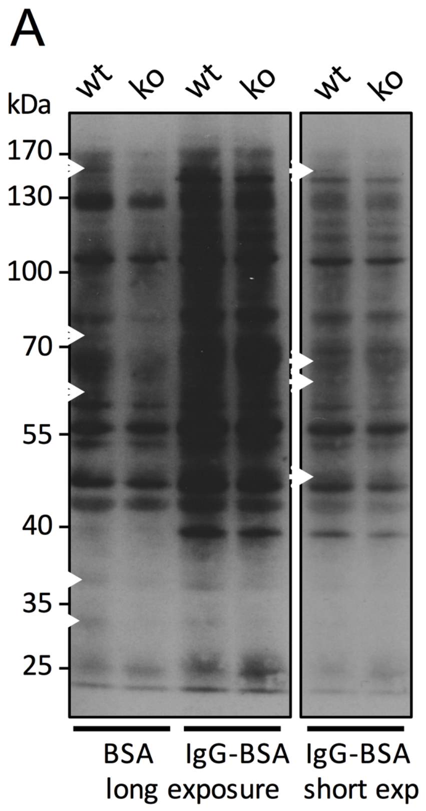

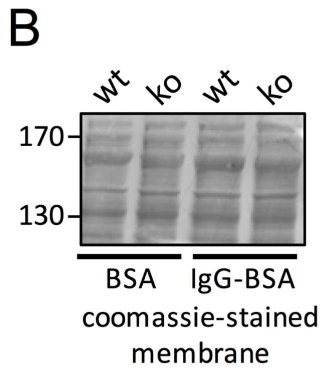

Supplemental figure 3. Ptpn22$^{-/-}$ neutrophils are hypoactivated following stimulation of immune receptors. *Ptpn22*$^{-/-}$ (ko) and matched wild-type control (wt) bone marrow derived neutrophils were allowed to adhere to immobilized ICs (IgG-BSA) or, as a control, BSA. Lysates were prepared and subjected to immunoblotting with an antibody specific for phosphotyrosine; two exposures are shown (A). The membrane was stained with coomassie brilliant blue to assess loading was equal (B).
